# Central lung adenocarcinoma in a young male mimicking pneumonia with nonrecurrent polyserous effusions of negative cytology: A case report

**DOI:** 10.1097/MD.0000000000039189

**Published:** 2024-08-02

**Authors:** Ayat A. Aljuba, Balqis Mustafa Shawer, Roa’a M. Aljuneidi, Safa Halman, Afnan W.M. Jobran, Mohammed Abdulrazzak, Orwa Al Fallah, Nidal E.M. Al Jebrini, Izzeddin A. Bakri, Yousef Abu Asbeh

**Affiliations:** a Faculty of Medicine, Palestine Polytechnic University, Hebron, State of Palestine; b Faculty of Medicine, Al Quds University, Jerusalem, Palestine; c Faculty of Medicine, University of Aleppo, Aleppo, Syria; d Radiology Department, Al-Ahli Hospital, Beit Jala, Palestine; e Oncology Department, Beit Jala Governmental Hospital, Beit Jala, Palestine; f Pathology Department, Makassed lslamic Charitable Hospital, Beit Jala, Palestine; g Thoracic Surgery Department, Al-Ahli Hospital, Beit Jala, Palestine.

**Keywords:** central mass, cytology, lung adenocarcinoma, pneumonia, polyserous effusions, young adult

## Abstract

**Introduction and Importance:**

Lung adenocarcinoma may resemble the clinical presentation of an infectious or inflammatory lung disease. The coexistence of lung cancer, and polyserous effusions is uncommon, which may cause a diagnostic challenge. However, any polyserous effusions at a young age must always be suspicious for malignancy.

**Case presentation:**

We report a case of 38-year-old male patient with polyserous effusions and pneumonia who was treated accordingly and showed clinical improvement with a significant reduction of pericardial and pleural effusions. Subsequent testing and a biopsy resulted in the histopathological diagnosis of an adenocarcinoma of the lung.

**Clinical Discussion:**

Nonrecurrent polyserous effusions in lung adenocarcinoma are uncommon, and negative cytology results may not exclude malignancy due to the moderate sensitivity of pleural and pericardial fluid cytology. Clinicians should remain vigilant for false-negative results, especially in younger patients. Malignancy should not be ruled out because pleural and pericardial fluid cytology have a sensitivity of 60% and 92%, respectively.

**Conclusion:**

Our case highlights the diagnostic challenges posed by atypical presentations of lung adenocarcinoma and emphasizes the importance of considering malignancy in the differential diagnosis of polyserous effusions, even when initial cytology results are negative. Clarifying the rationale for this study enhances its relevance and impact.

## 1. Introduction

Lung cancer is the most common cancer leading to death worldwide. It is generally divided into non–small-cell lung cancer (NSCLC), 80%, and small cell lung cancer.^[1]^ Approximately 70% of NSCLC, like adenocarcinoma, is diagnosed in the late or metastatic stage.^[2]^ Most patients diagnosed with lung cancer are 65 or older, but 3.5% of them are 45 years old or younger.^[3]^

Computed tomography (CT) can reveal a variety of lung adenocarcinomas, such as a single nodule or mass, a thin-walled cystic lesion, localized or widespread parenchymal consolidation, or multifocal lesions.^[4]^ Due to the difficulty in differentiating lung adenocarcinoma from pneumonia when it presents as parenchymal consolidation, diagnosis of the condition is frequently delayed.^[4]^

Polyserous effusions are defined as fluid accumulation in 2 or more serious cavities, which is quite rare in adenocarcinoma patients and frequently misdiagnosed with other etiologies.^[5]^ However, any polyserous effusions at a young age must always be suspicious for malignancy. Ten percent of cancer patients get pericardial effusion, with lung cancer being the most common cause.^[6]^ However, pleural effusion and metastatic ascites occur in 7% to 15% and 2.7% to 16% of lung cancer patients, respectively.^[7]^

In our article, we report a case of a 38-year-old male who presented as a complicated pneumonia case with nonrecurrent polyserous effusions and negative pleural and pericardial cytology, which was attributed to central adenocarcinoma of the lung.

## 2. Case presentation

A 38-year-old heavy smoker male with no pertinent medical history presented to the internal clinic with colicky right-sided and epigastric abdominal pain of 3-day duration that increased with eating and was not relieved with H2 blockers but partially relieved by Ibuprofen, associated with abdominal distension, sweating, nausea, and vomiting of clear fluid without blood. He also reported generalized fatigue, feverish sensation, anorexia with recent weight loss.

Symptoms became progressive and developed dyspnea and cough without hemoptysis. The patient denied any history of chest pain, palpitations, cyanosis, leg swelling, dizziness, or other symptoms. He also denied chest, abdominal trauma and recent travel. He had a family history of lung cancer in his paternal uncle at the age of 35 years, in addition to a late-onset lung cancer in his grandmother. His aunt was diagnosed with breast cancer at the age of 45 years, too.

**Figure 1.**
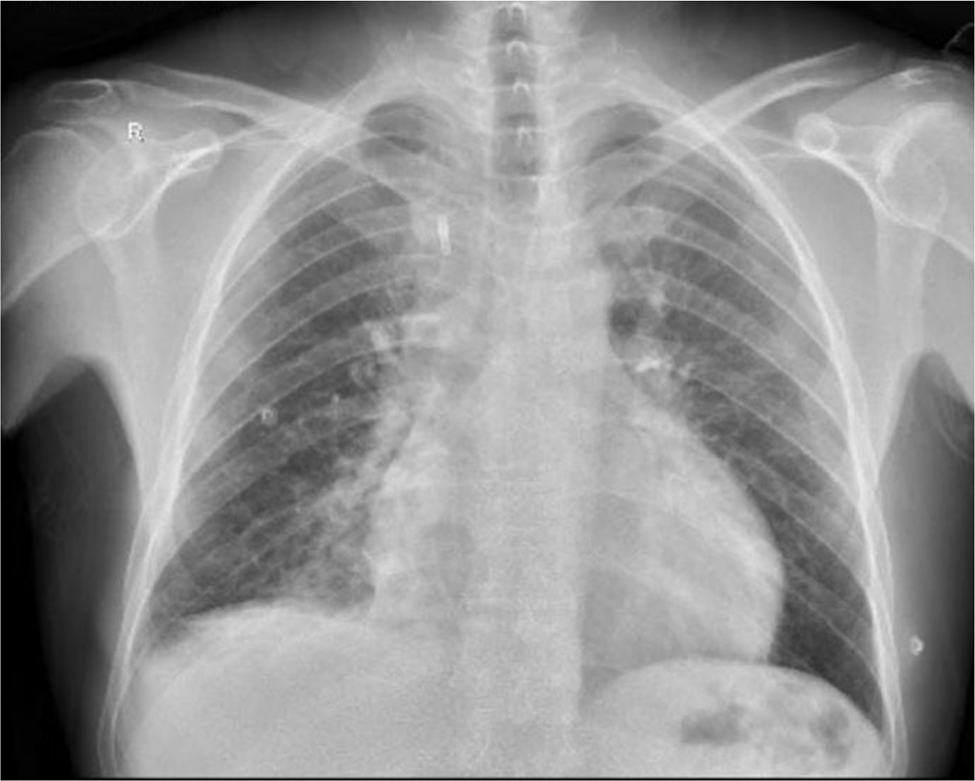
Chest X-ray (CXR) shows retrocardiac consolidation of the right lower lobe of lung, enlargement of the cardiac outline with water bottle sign suggesting pericardial effusion and obliteration of right costopherinc angle denoting moderate right-sided pleural effusion.

The physical examination was unremarkable except for diffuse tenderness and guarding of the abdomen, more prominent in the right upper and lower quadrants, accompanied by rebound tenderness. Chest X-ray showed retrocardiac consolidation of the right lower lobe of lung, enlargement of the cardiac outline suggesting pericardial effusion and moderate right-sided pleural effusion (Fig. 1).

**Figure 2.**
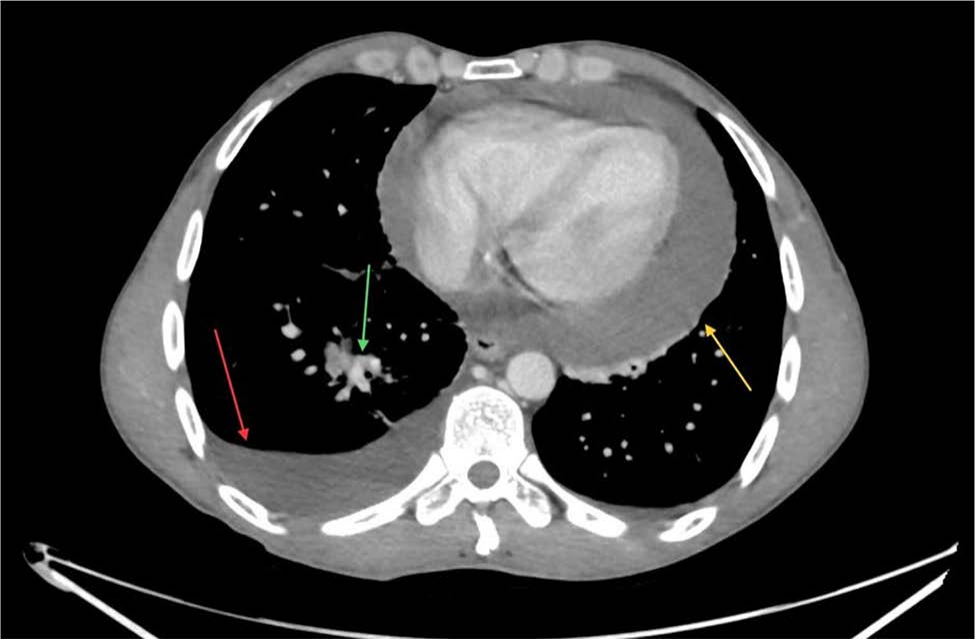
Chest CT scan, transverse view, illustrating a pericardial effusion (yellow arrow), right pleural effusion (red arrow), and right lower lobe lesion (green arrow). CT = computed tomography.

Chest and abdominal CT with intravenous contrast revealed ascites, right lower lobe lesion suggesting consolidation, associated pleural and pericardial effusion (Fig. 2). Subsequently, echocardiography detected a large pericardial effusion with significant right ventricular compression and normal left ventricular size and function.

Abdominocentesis and biochemistry showed a yellow, turbid fluid with increased lactate dehydrogenase, normal glucose, total protein and albumin. Pleural tapping and biochemistry analysis revealed an exudative fluid. Pericardiocentesis showed a bloody, turbid aspirate. The cultures of the 3 fluid samples showed no evidence of bacterial or fungal growth. Peritoneal, pleural, and pericardial fluid analysis are shown in Table 1.

**Table 1:** Peritoneal, pleural and pericardial fluid analysis with serum values.

Test	Peritoneal fluid	Pleural fluid	Pericardial fluid	Reference range	Serum	Reference range	Unit
Glucose	108	105	37 (decreased)	70–110	106	60–200	mg/dL
LDH	121 (increased)	112 (increased)	1156 (increased)	0–1	168 (decreased)	250–450	U/L
Total protein	3.7	2.7	5.2 (increased)	<4.1	6.1	6–8	g/dL
Albumin	2.4	1.8 (decreased)	3.3	2–2.7	3.9	3.8–5.5	g/dL

LDH = lactate dehydrogenase.

Samples of pleural and peritoneal fluid and the pericardial window were sent for cytology and pathology. Considering the diagnosis of pneumonia, he was given colchicine, ibuprofen, metronidazole, and levofloxacin. A few days later, the patient’s symptoms improved dramatically.

The cytology of the peritoneal fluid was interpreted as showing macrophages, neutrophils, and occasional lymphocytes with reactive mesothelial cells and being negative for malignancy. The cytology of the right pleural fluid revealed few reactive mesothelial cells, many lymphocytes, few neutrophils, and the absence of cancer cells. A pericardial window under echocardiography-guidance illustrated mild chronic inflammation with no evidence of malignancy.

**Figure 3.**
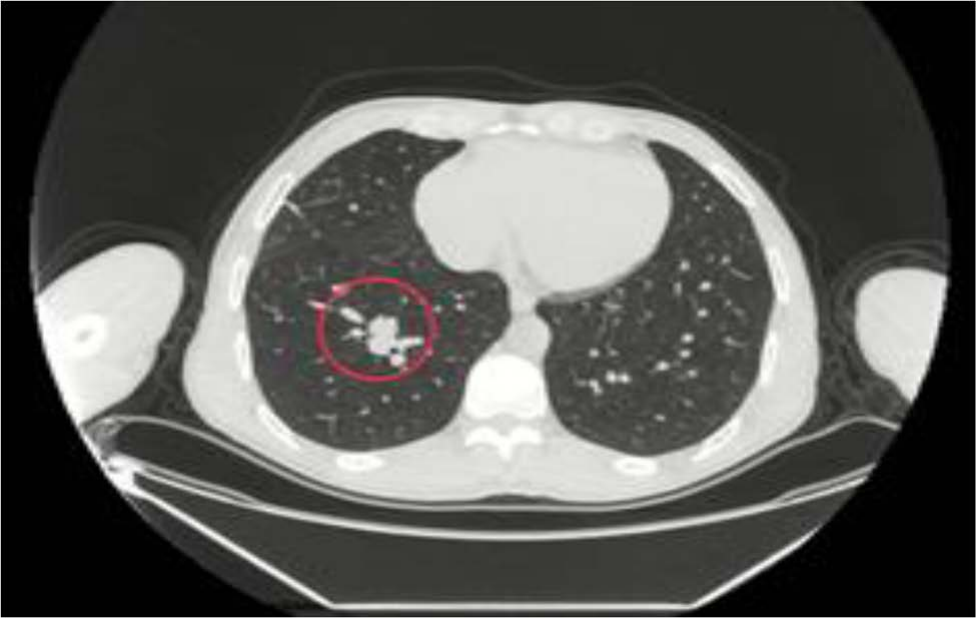
Chest CT scan, transverse view, demonstrating a spiculated mass in the right lower lobe of the lung (red circle). CT = computed tomography.

**Figure 4.**
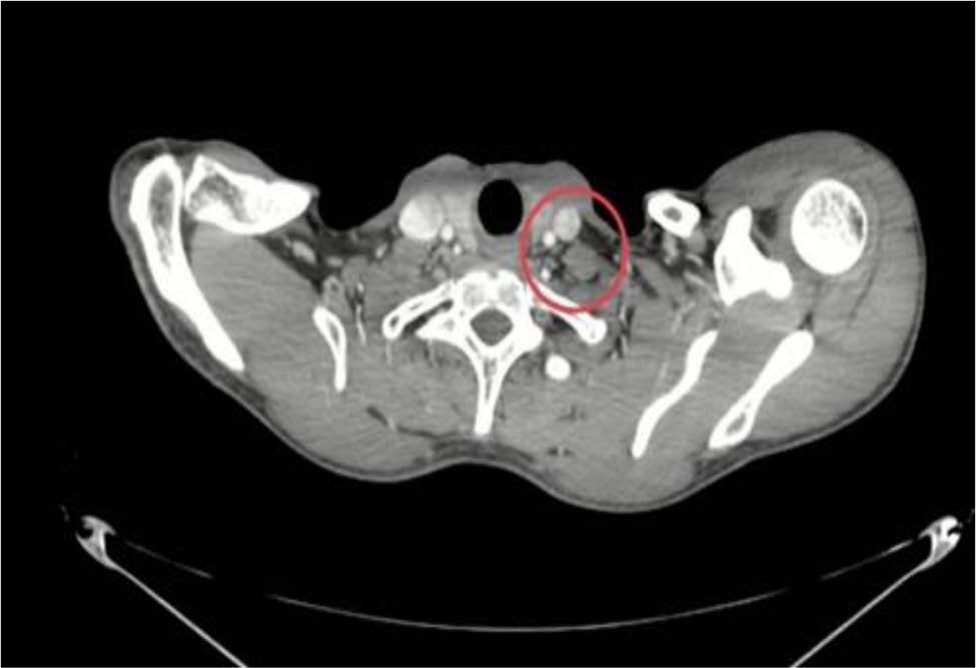
Neck CT scan, transverse view, illustrating an enlarged left supraclavicular lymph node (red circle). CT = computed tomography.

A follow-up CT scan revealed resolution of almost all of the pleural effusion, with significant reduction of pericardial effusion but persistence of the spiculated soft tissue enhancing lesion measuring about 2.1 cm in the right lower lobe (Fig. 3) associated with hilar and supraclavicular lymphadenopathy (Fig. 4).

**Figure 5.**
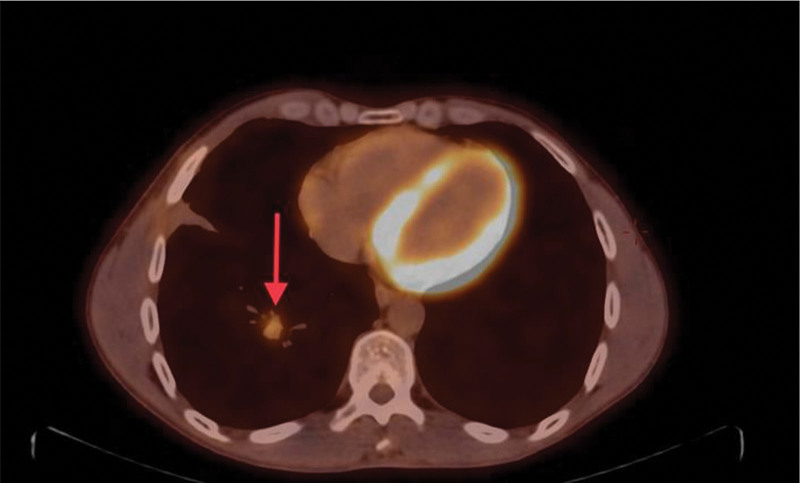
Nodular shadow with elevation of metabolism in the posterior basal segment of the right lower lobe of the lung on transverse view (red arrow).

**Figure 6.**
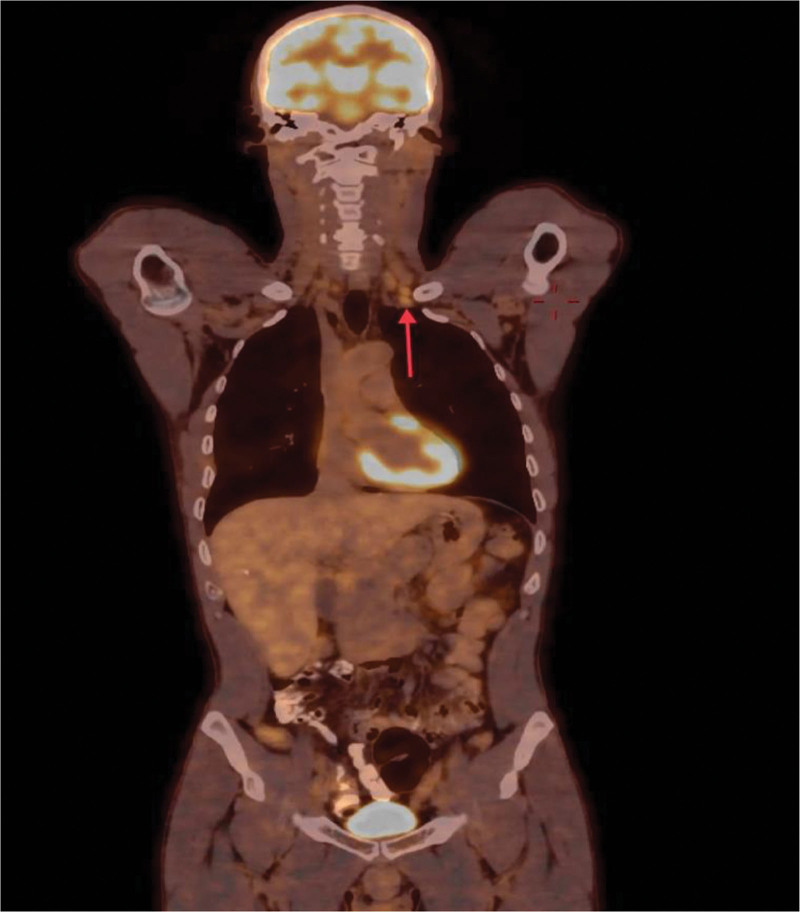
PET scan revealing a hypermetabolic prominent-sized left supraclavicular lymph node on coronal view (red arrow). PET = positron emission tomography.

**Figure 7.**
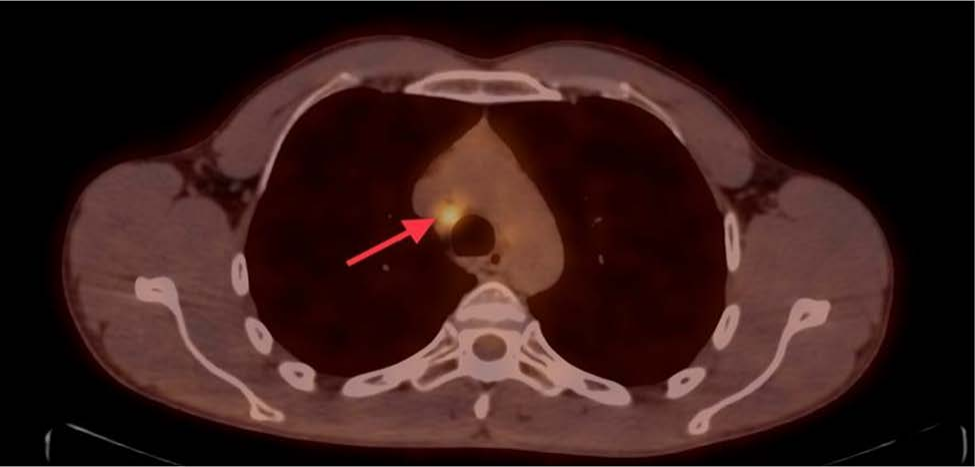
PET scan showing hypermetabolic mediastinal lymph nodes on transverse view (red arrow). PET = positron emission tomography.

For further evaluation, a whole-body positron emission tomography scan was performed that demonstrated a hypermetabolic potentially malignant right pulmonary nodule in the posterior basal segment of the lower lobe (Fig. 5), bilateral prominent-sized supraclavicular lymph nodes, the left being larger (Fig. 6), and prominent right mediastinal and hilar lymph nodes (Fig. 7). Magnetic resonance imaging of the brain was performed with no evidence of metastatic disease.

**Figure 8.**
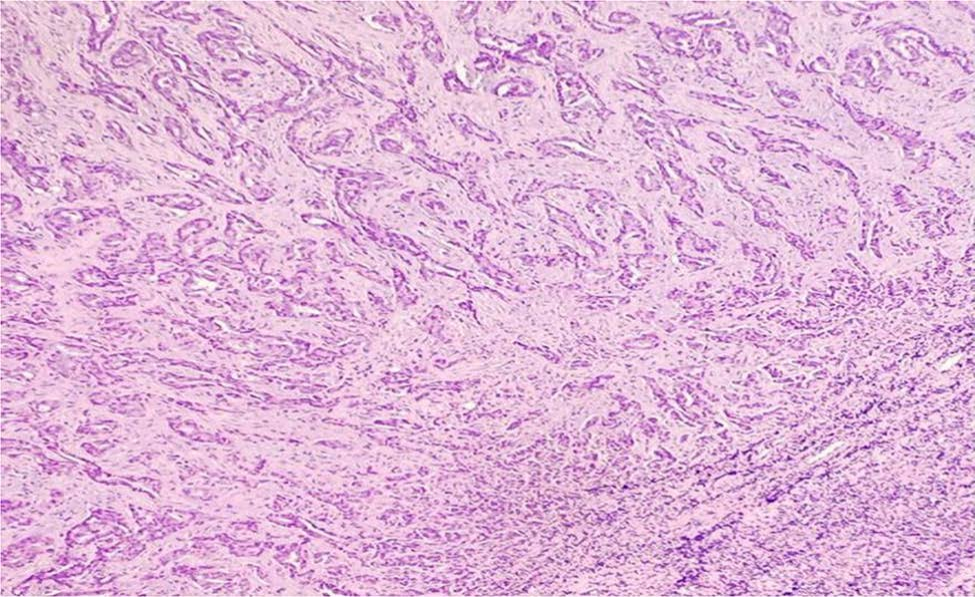
Hematoxylin and eosin stain of left supraclavicular lymph node showing metastatic moderately differentiated adenocarcinoma “acinar” of lung origin.

**Figure 9.**
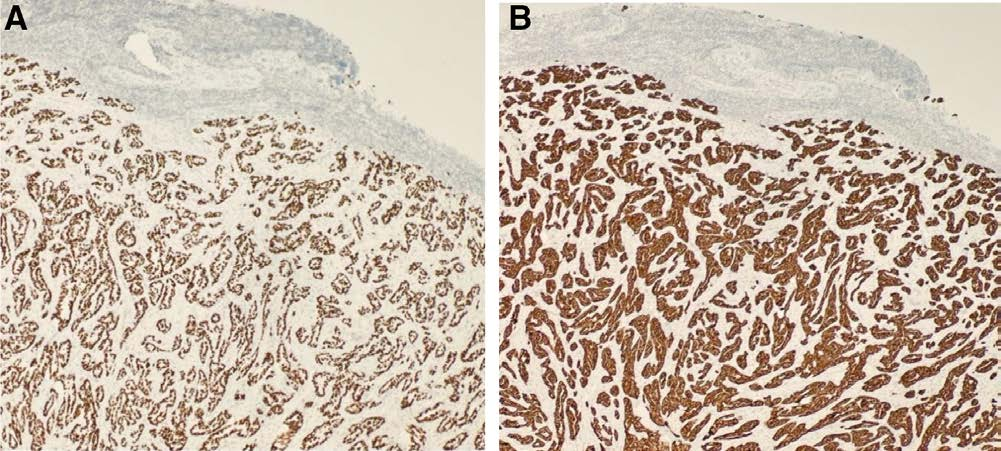
(A) TTF-1 immunostain “strongly positive “nuclear.” (B) CK 7 immunostain “strongly positive.” CK 7 = cytokeratin-7, TTF-1 = thyroid transcription factor.

An excisional biopsy of the supraclavicular lymph node confirmed Metastatic moderately differentiated adenocarcinoma of lung origin (Fig. 8). Immunohistochemical stains were positive for thyroid transcription factor (TTF-1) and cytokeratin-7 (CK7), which is consistent with primary pulmonary adenocarcinoma (Fig. 9A, B).

**Figure 10.**
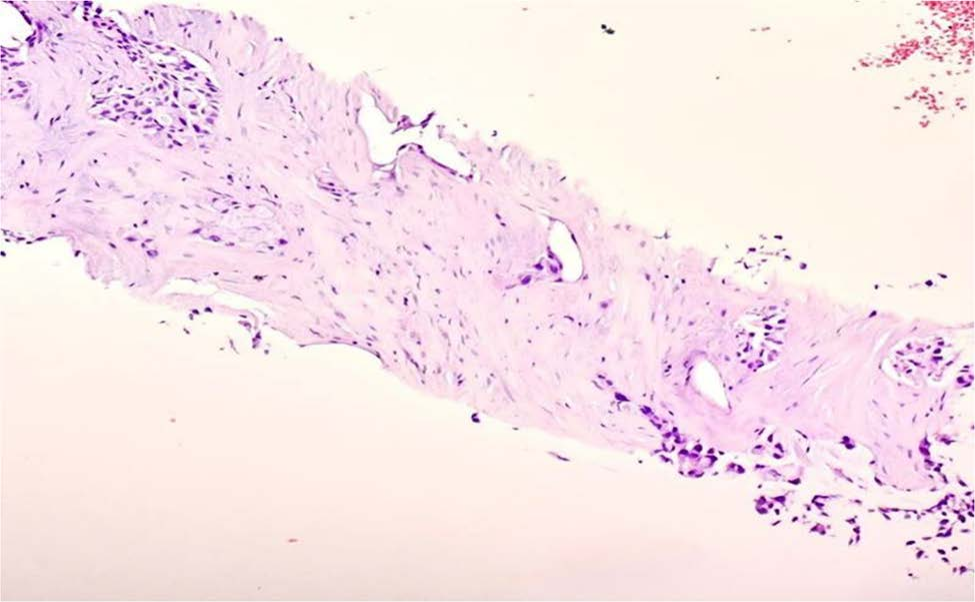
Biopsy of right lower lung mass with hematoxylin and eosin stain showing invasive moderately differentiated adenocarcinoma.

A puncture biopsy of the right lung mass was performed with CT guidance, and histopathology confirmed it as invasive, prominent right mediastinal and hilar lymph nodes (Fig. 10).

Thus, he was diagnosed with stage IIIC lung adenocarcinoma. Molecular testing revealed that the tumor was negative for epidermal growth factor receptor mutations, anaplastic lymphoma kinase gene, cROS oncogene 1 (ROS1), and programmed cell death ligand 1. Accordingly, the patient was started on chemoradiation therapy.

## 3. Discussion

In our case, lung adenocarcinoma presented a case of pneumonia with polyserous effusions that responded to treatment and almost completely resolved without recurrence. In contrast to the literature, a high likelihood of symptomatic, ipsilateral pleural fluid recurrence within 100 days of the initial thoracentesis exists in patients with advanced metastatic NSCLC and large unilateral pleural effusion.^[7]^ According to these facts, this presentation is unique, and pneumonia with polyserous effusions should always be suspicious for malignancy, especially at a young age. Moreover, the association between central lung adenocarcinoma and pleural, pericardial, and ascites effusions has been well-documented in the literature.

Additionally, compared to our patient, who presented with advanced lung cancer at the young age of 38 years, only 0.9% of patients in the 35 to 44 age group are diagnosed with lung and bronchus cancer. Lung and bronchial cancers are most often diagnosed in adults aged 65 to 74 years.^[8]^

Tissue histology provides a conclusive diagnosis of lung carcinoma. According to studies, pleural fluid cytology performed following the initial thoracocentesis had a 60% sensitivity for detecting lung cancer.^[9]^ Repeating this procedure causes it to rise to 75%. As a result, it is not appropriate to diagnose lung cancer merely based on the pleural cytology or biopsy’s lack of malignant cells.^[8]^ In addition, pericardial fluid cytology had a sensitivity of 92.1% in diagnosing cancer, whereas pericardial biopsy had a sensitivity of 55.3%.^[8,10]^

In this case, both the pleural and pericardial effusion cytology and pericardial biopsy were negative for malignancy. However, malignancy was not ruled out because pleural biopsy results in patients with lung cancer can be negative. Pleural cytology is negative in two-thirds of lung tumors.^[9]^

Hence, additional investigations should be performed, including thoracotomy, medical thoracoscopy, video-assisted thoracic surgery, and image-guided cutting needle biopsy.^[9]^ Video-assisted thoracic surgery was excluded because of advanced disease, and since thoracotomy is very invasive, CT-guided biopsy and bronchoscopy were the only remaining options.^[10]^

Here is a challenge to be mentioned: the lung lesion was located centrally, so it was not easily accessible by CT-guided biopsy or by bronchoscopy. However, a hard decision to proceed with a CT-guided biopsy was made since it was the only available option, provided that a thoracic surgeon was on the scene in case any bleeding or complications happened. Even though this was very challenging, the CT-guided biopsy was done without any complications.

There is some debate about the stage of this cancer; oncologists view it as stage IIIC because there is no proof about the malignant origin of the polyserous effusions. On the other hand, the thoracic surgeon’s point of view is stage IV because there is no explanation for these polyserous effusions at such a young age, which were not attributed to an inflammatory or infectious origin. Especially when ascites, pleural and pericardial effusions are present in lung cancer patients, it means the illness is advanced and the median survival time is short.^[5]^ Consequently, the patient was treated as a case of stage IIIC and started with chemoradiation.

In our study, we acknowledge several limitations that may have affected the scope and findings of our research. Firstly, the retrospective nature of the case report limits the ability to draw definitive conclusions about the association between polyserous effusions and lung adenocarcinoma. Additionally, the small sample size of our study, limited to a single case, restricts the generalizability of our findings to a broader population. Furthermore, the diagnostic challenges encountered in this case, including the negative cytology results and the difficulty in accessing the centrally located lung lesion for biopsy, highlight the complexities and limitations inherent in diagnosing and staging lung adenocarcinoma with polyserous effusions. These limitations emphasize the need for further research and larger studies to better understand the clinical implications and management of lung adenocarcinoma presenting with polyserous effusions.

## 4. Conclusion

This case described a patient in his fourth decade of life presenting with complicated pneumonia and nonrecurrent polyserous effusions with negative cytology, which is surprisingly uncommon. Although pleural fluid cytology and biopsy are still used in most algorithms for detecting lung cancer, their poor sensitivity and high incidence of false-negative findings should worry clinicians. Clinicians should be aware of the unusual presentation of lung adenocarcinoma since it frequently results in misdiagnoses of infectious and inflammatory lung disorders, making a thorough workup is essential.

## Author contributions

**Conceptualization:** Ayat A. Aljuba, Balqis Mustafa Shawer, Roa’a M. Aljuneidi, Safa Halman, Mohammed Abdulrazzak.

**Data curation:** Ayat A. Aljuba, Balqis Mustafa Shawer, Roa’a M. Aljuneidi, Safa Halman, Afnan W.M. Jobran, Orwa Al Fallah, Nidal E.M. Al Jebrini, Izzeddin A. Bakri, Yousef Abu Asbeh.

**Project administration:** Ayat A. Aljuba.

**Writing—original draft:** Ayat A. Aljuba, Balqis Mustafa Shawer, Roa’a M. Aljuneidi, Safa Halman, Mohammed Abdulrazzak, Orwa Al Fallah.

**Writing—review & editing:** Ayat A. Aljuba, Balqis Mustafa Shawer, Roa’a M. Aljuneidi, Safa Halman, Afnan W.M. Jobran, Mohammed Abdulrazzak, Orwa Al Fallah, Nidal E.M. Al Jebrini, Izzeddin A. Bakri, Yousef Abu Asbeh.

**Resources:** Balqis Mustafa Shawer, Roa’a M. Aljuneidi, Afnan W.M. Jobran, Mohammed Abdulrazzak, Yousef Abu Asbeh.

**Validation:** Safa Halman, Afnan W.M. Jobran, Mohammed Abdulrazzak, Orwa Al Fallah, Izzeddin A. Bakri, Yousef Abu Asbeh.

**Supervision:** Afnan W.M. Jobran, Yousef Abu Asbeh.

**Investigation:** Nidal E.M. Al Jebrini, Izzeddin A. Bakri.
